# The Nature and Treatment of Sea Sickness

**Published:** 1848-01

**Authors:** F. Willis Fisher


*The nature and treatment of Sea Sickness.* By F. WILLIS FISHER, M. D.—If we were to judge of a disease from the painful sensations that it causes, rather than from the danger it involves, we should be forced to class sea sickness in the rank of the scourges of humanity. This affection kills no one, but causes those affected by it to suffer severely. Many marine officers have been compelled to give up the life they had chosen, because the habit of navigation could not relieve them from the occurrence of nausea every time the sea became rough and agitated. Some persons have renounced revisiting their country and their families, sooner than expose themselves again to what they suffered from sea sickness on their first voyage. Every scholar knows that Cicero preferred giving his head to the assassins of the triumviri, rather than remain a few moments longer a prey to the pain of sea sickness on the vessel which bore him far from the shores occupied by his enemies. A morbid state, capable of imposing the sacrifice of all that man holds most dear, the sacrifice of ambition, that of the natural affections, and even of life, surely merits the attention of the physician. Upon the nature of sea sickness, and the rational means to employ with the view to avoid and combat it, nothing positive is as yet known ; a proof of which lies in the diversity of opinions on this subject. We do not think that the true theory of it has as yet been given.

Nearly all writers have considered the affection in a reverse sense of what is really the case :—for example, in attributing sea sickness to a sanguineous congestion of the brain ; or, assigning it a cause in fact incapable of producing it, in referring it to shocks or agitations that are communicated to the intestines by the motion of the vessel. To form an estimate of these two opinions, the experience and theory of M. Pellarin during his service as marine surgeon, seem deserving of attention, as approaching nearer the true cause and theory of this disagreeable affection. The invasion of sea sickness, far from being accompained by the ordinary symptoms of congestion, a flushed countenance, vascular turgescence, full pulse, sensation of heat and tension in the cranium, throbbing of the temporal arteries, the eyes brilliant and injected, &c., is rather characterized by the opposite state—a paleness of the face and hands, a retreat of the blood from the surface, a depressed pulse, general hyposthemia, a dull, glassy eye when the affection is at its highest point. M. Pellarin has never observed any of the accidents of cerebral hyperemia in individuals affected by sea sickness. If during great efforts of vomiting, the blood flows to the head for the moment and colours the face, it is only the instantaneous result of these efforts ; the paleness soon reappears, with all the other characters of the anæmic state, just as it happens when one is under the influence of tartar emetic, taken in such a dose as to produce vomiting.

Another consideration, which ought still more to remove the idea of the sanguineous cerebral congestion, is that one suffers less when lying down, than when standing ; and less still, if instead remaining simply in a horizontal position, he has his head lower than the rest of the body.

As to the explanation which would make this affection to depend upon the shocks impressed upon the intestinal mass, this resists examination no better than the first. The trotting of a horse shakes the bowels much more than the pitching and rolling motion of a vessel, yet it never causes anything that resembles sea sickness. Sickness from riding in a carriage is of the same nature as the last; it is like the disagreeable sensations caused to some persons by swinging. This sickness is sooner felt in a carriage suspended with springs, than in a hard jolting cart, which shakes the organs much more than an easy carriage. One may make upon himself the experiment of the mechanical shock impressed on the intestinal mass, by agitating the floating portion of the abdominal viscera with his hands, by giving them successive impulsions, either from below upwards, or in any other direction, and he can never cause by these manœuvres anything analogous to sea sickness. Compression, a kind of kneading of the stomach when distended by food, may sometimes cause the expulsion of a portion of its contents, but it does not resemble that strange uneasiness and profound prostration which characterize sea sickness.

The other explanations ordinarily given for sea sickness—such as the sanguineous congestion of the brain, the shaking of the abdominal viscera; that this affection has a cause altogether nervous, depending principally upon the nerves that excite the epigastric and abdominal viscera, &c., throw no light upon the question.

M. Jobard, of Brussels, without doubt, has reason in saying that the essential cause of sea sickness is purely mechanical. However, he goes too far when he adds that the odour of the vessel does not the least contribute to excite it. Although this state of uneasiness may be caused by the movements of the vessel, yet it is not less true, that whatever excites repugnance, the odour of the tarry materials, the emanations that come from the hold and other low parts of the vessel, the sight of persons vomiting, all these impressions second the nauseous influence of the mechanical cause of sea sickness, and tend to produce it, from sympathy. Moreover, the proofs that sea sickness depends essentially upon the motions of rolling and pitching, are so evident that it is not worth the trouble to cite them. The nausea arises under the influence of these movements, and is generally proportioned to their extent and quickness. It is felt less in the centre of the ship, near the foot of the mainmast, because the double motion is less there than at the edge, especially at the extremities where the pitching is most considerable. In a hammock or frame suspended so as to have as little friction as possible, which rests always in the direction of the perpendicular, and consequently not subject to the different inclinations of the vessel, one nearly escapes being sick. The production of vertigo and nausea which precedes the vomiting, may in part be imputed to the impressions resulting from the sight of objects which appear to rise and fall alternately in relation with the vessel. Regarding the horizon continually oscillating and moving, or the steerage of the vessel, or the water that seems to fly along its sides, is sometimes sufficient to determine the crisis of sea sickness. From this follows the opinion that it is especially by the eyes that sea sickness effects the economy. Nevertheless, as some have pretended, the visual impression is not the essential cause of the nausea, for it is equally experienced in the obscurity of night and by blind persons.

M. Pellarin has not remarked so striking a difference as M. Jobard, and many others, between the influences of rising and falling, and he affirms that when one is forward, the crisis of the nausea takes place at the moment this extremity rises. Whatsoever it may be, M. P. is disposed to admit what a marine officer recently told him. It is in the rising motion or ascension that the nausea commences, but it is in that of descending that the nausea is exasperated and acquires all its intensity. The following is the theory of M. Pellarin. Sea sickness ought to be attributed to the trouble caused in the circulation of the blood by the alternate movements of inclination that the ship undergoes; either lateral, rolling; or antero-posterior, pitching. This trouble has for a result, not to congest the brain, as Wollaston pretends, but, on the contrary, to deprive it of a sufficient quantity of blood for the normal stimulation of the nervous centre. That which is experienced in sea sickness is in fact analogous to what often happens in arresting the flow of blood in persons who are bled while sitting or standing, and who at the time they faint are taken with a disposition to vomit and really do vomit. M. P. does not deny that by reason of the general diminution of the circulation there may be a stagnation of the venous blood in the cerebral sinuses, but it is especially in the want of a sufficient excitation of the nervous centres by the arterial blood that the primordial phenomenon of sea sickness seems to consist. Observe a person seized by sea sickness; his face becomes pale, his extremities cold, his nails turn blue as at the debut of intermittent fever. What he experiences resembles much the effects produced by the smoking of the pipe or the segar, on persons who are not accustomed to smoke. The pulse becomes small, and there is an extreme prostration of the intellectual and physical faculties. There is a hyposthenic influence in both cases, by the narcotic action of the tobacco in one case; by the diminution of the circulatory force of the blood in the other. What individuals best resist sea sickness? Very young children, those who are at the breast, in whom the heart is relatively more voluminous, and the circulation more active than in adults, are not sensibly incommoded by the affection. Without being wholly exempt, animals experience it less than men, because with them the brain is nearly in the same horizontal plane as the heart, and it is not rare to see the poultry in their first voyage, present nearly all the signs of this affection, almost to vomiting when the sea is rough. Among the adult passengers, those who take the least exercise, and who go on deck in the breeze the least, remain the longest under the influence of sea sickness. And among persons equally habituated to sea life, those who by their functions or rank have the least corporeal activity, are more liable to return of nausea than the common sailor who works the vessel, who mounts the masts and yards, and is exposed to more tedious movements than those on deck. Dulness of spirits and lassitude, a cold drizzling rain that cools the skin, and diminishes the circulation, are predisposing causes. Towards the close of sea sickness, when the nausea and vomiting begin to leave some respite, one is inclined to somnolence, as after hemorrhages. Is it not by a sedation of the same kind that infants are quieted and put to sleep by rocking them? In fine, M. P. concludes that whatever raises the force and accelerates the rhythm of the circulation, prevents or diminishes the liability to this affection. Strong and frequent respirations act thus, according to the testimony of M. Arago, who warded off sea sickness until the fatigue of the respiratory muscles obliged him to renounce this prophylactic means. M. Jobard and many others have recommended a girdle which compresses the abdomen at the base of the chest. This in truth alleviates, but not because it confines the intestines, but because, it contributes to push the blood towards the brain. It acts in the same manner as a person lying down with the the head low, a position that is sufficient to dissipate the nausea of persons affected by syncope, or that after bloodletting, which state presents a striking analogy to sea sickness. Moreover, a proof that compression of the chest and abdomen is not a sovereign remedy, is, that corsets do not prevent women from being affected by it. In these two comparative states (hypothymic nausea, after bloodletting, and maritime nausea,) the impression of a sharp breeze is equally favourable, and the first symptoms have been sometimes overcome by going on deck, and receiving the direct action of a brisk current of air. To verify the theory of M. Pellarin, if those who are placed in circumstances that cause sea sickness should have large cuppings from the lower limbs, they would experience the first attacks sooner, as in this case there would be two concurring causes to deprive the brain of the normal afflux of blood that it ordinarily received. Another mode of verification that M. P. has not employed, is auscultation applied to the large vessels of the neck; we are inclined to think that the *bruit de soufflet* ought to be heard in individuals who are affected by sea sickness, as well as with chlorosis.

M. P. recognizes an analogy between the nausea produced by the motions of a vessel, and the nausea and vomiting of women during the first months of pregnancy; that is, at an epoch when the womb becomes the centre of a sanguineous afflux, and consequently diverts from the brain a portion of the vivifying liquid it received. Many women have declared that nothing resembled more the nausea of the comencement of their pregnancy than that they experienced the first few days at sea. Another circumstance which strengthens this theory is, that generally pregnant women are rarely taken with vomiting while they remain in bed, and, on the contrary, often so taken, when they change the horizontal to an upright position. Why are women more nervous? why have they odd tastes and irresistible desires, during the period of pregnancy? Is it not because the nervous system is at this time less supplied with blood, and that the blood, as every one knows, is the moderator of the nerves. A similar cause produces the greatest susceptibility among women during the menstrual period. To cite an example—a lady, who had never been sea sick during many voyages, experienced it severely in crossing the English Channel when she had one of her periodic evacuations.

To resume the conclusions. First, the sickness produced by the sea, by riding in carriages, by swinging, are all phenomena of the same nature, determined essentially by the influence exercised on the circulatory march of the blood in the movements that the body undergoes under these different circumstances. Second, this influence has its principal affect in diminishing the ascending force of the excitory liquid in the aorta and the arteries branching from it; from this results a hyposthenic state of the brain by anemia or hypohemia. Third, the insufficient excitation of the cerebral organs determines, by sympathy, spasmodic contractions of the diaphragm, vomitings—which have a particular tendency to reconvey the blood which is wanting towards the nervous centre. These efforts are a crisis which takes place in a conservative end. They manifest themselves not only in sea sickness, but in many other circumstances where the brain becomes suddenly deprived of its normal supply of blood; for example, in persons not affected by phlegmasia who are bled.

*Treatment.*—There are two orders of means to be employed. The first consists in removing one's self as much as possible from the cause, i. e., from the motions of the vessel, in remaining in a recumbent position, in a hammock suspended without sensible friction at its points of attachment. The second has for an end to combat the effects of the cause on the organism. It acts especially to this end in stimulating the circulatory function by all the agents susceptible of increasing its energy. Thus, a tonic regimen, active corporeal exercise for some days previous to embarkation. At sea, if the weather permits, one ought to keep on deck, in the breeze, make large inspirations, walk quickly and until he perspires or is fatigued; or, better still, to engage in some hard exercise, even with the sailors in working the vessel. Hard work, that which requires great muscular effort, is the surest prophylactic against sea sickness. The girdle has also its advantages in contributing to force the blood towards the head, and perhaps in seconding the contractile force of the heart. Before the manifestation of the nausea, warm and exciting drinks are favourable. Thus coffee, tea with the addition of a little brandy, may give a greater disposition to resist it, in stimulating the circulation and maintaining a diaphoretic state of the skin. Among the medicines, those which have an analogous effect on the economy may be administered with advantage, such as opium, saffron, acetate of ammonia, &c. When the sickness is declared, recourse is only to he had in the palliatives; lemons, exciting aromatics, relieve some persons; also the horizontal position, especially with the head low, in a hammock or bed suspended like a compass. But if one wishes to shorten the duration of the nauseous influence of the sea and diminish the tribute he must pay to a nautical acclimation, he must struggle with all his energy against the tendency to inaction.

*Therapeutic employment of sea sickness.*—A cause which determines in the economy so great a commotion as sea sickness, without leaving any unhappy consequences, as a therapeutic agent, merits more attention than has been given it. M. Pellarin thinks that it may be possible to obtain from it valuable results in many acute and chronic affections. This observation was familiar to the ancients. We read in Pliny, "Vomitings, produced by the motion of a vessel, act as a salutary remedy in many diseases of the head, eyes, chest, and in all affections for which hellebore is given." In more modern times, Esquirol and Blanche have judiciously advised its employment in cases of recent mania. But in the few attempts that have been made, there has happened, what might have been easily foreseen, from the true theory of maritime nausea, that the maniacs, highly excited, have not been affected by sea sickness, whilst the physicians who accompanied them have been a prey to it during the whole voyage. From the knowledge already acquired of the nature and etiology of sea sickness, there seems nothing in the way to second, to aggravate voluntarily its influence in a curative end. Even an apparatus might be made to produce all the effects of rolling and pitching, without the necessity of a sea voyage. By reason of the powerful sedative and hyposthenic influence of sea sickness, may we not draw from its employment the greatest advantages, not only in acute cerebral affections, but also in certain pneumonias, pleurisies, and, finally, in a great number of inflammatory diseases?—*Boston Med. and Surg. Journ.*

